# Letrozole administration during ovarian stimulation: intrafollicular concentrations, effects on steroidogenesis and pregnancy outcome—a secondary analysis

**DOI:** 10.1007/s10815-026-03890-6

**Published:** 2026-05-04

**Authors:** Elisabeth Minke Elfrink, Malene Louise Johannsen, Kenneth Munk Pedersen, Liv la Cour Poulsen, Mengxue Zheng, Marie Louise Grøndahl, Anders Hay-Schmidt, Bjarne Styrishave, Claus Yding Andersen

**Affiliations:** 1 Drug Toxicology, Metabolism and Analysis Group, Department of Pharmacy, Faculty of Health and Medical Sciences, University of Copenhagen, Copenhagen, Denmark; 2 The Fertility Clinic, Herlev Hospital, Herlev, Denmark; 3 Department of Odontology, Faculty of Health and Medical Sciences, University of Copenhagen, Copenhagen, Denmark; 4 Reproductive Medicine Centre, Yantai Yuhuangding Hospital, Shandong Province, China; 5 Institute of Clinical Medicine, Faculty of Health and Medical Sciences, University of Copenhagen, Copenhagen, Denmark; 6 Department of Urology, Copenhagen University Hospital - Herlev and Gentofte Hospital, Herlev, Denmark

**Keywords:** Fertility treatment, Letrozole, Follicular fluid, Sex steroids, Pregnancy

## Abstract

**Purpose:**

To determine intrafollicular concentrations of letrozole (LTZ) and key steroids in women undergoing ovarian stimulation (OS) with or without LTZ co-treatment, and to explore associated clinical outcomes.

**Methods:**

Follicular fluid (FF) collected at oocyte pickup from 30 women participating in the RIOT-B study, a randomized controlled trial comparing OS with recombinant FSH (150 IU/day) combined with either LTZ (5 mg/day; *n* = 15) or placebo (*n* = 15). Concentrations of LTZ and steroid were quantified by LC–MS/MS. Associations between FF composition and clinical outcomes were evaluated.

**Results:**

At oocyte pickup, mean FF LTZ concentration was 144 ± 12 nmol/L (nM) (range 51–270). FF 17β-estradiol (E_2_) concentrations were similar between groups (≈900 nM), whereas testosterone, androstenedione, DHEA, and 17OH-progesterone (17OH-P_4_) were markedly elevated in the LTZ group (*p* < 0.001–0.0001). In contrast, P_4_ levels remained unchanged. Among LTZ-treated women, higher FF concentrations of LTZ and testosterone were significantly associated with failure to conceive. No relationship was observed between FF LTZ levels and BMI or gonadotropin dose.

**Conclusions:**

Intrafollicular estrogen levels remain preserved during LTZ administration, accompanied by pronounced androgen accumulation and elevated 17OH-P_4_. These findings suggest complex, cell-specific effects of aromatase inhibition on follicular steroidogenesis and raise questions regarding the optimal dosing and mechanistic rationale for LTZ co-treatment in assisted reproduction.

**Supplementary Information:**

The online version contains supplementary material available at 10.1007/s10815-026-03890-6.

## Introduction

The purpose of ovarian stimulation (OS) with exogenous gonadotropin administration is to augment the number of preovulatory follicles and oocytes for fertility treatment [1]. This is invariably linked to supra-physiological concentrations of 17β-estradiol (E_2_), which is mainly produced by granulosa cells (GCs) through the aromatase-mediated conversion of theca cell-derived androgens. This pathway is central for paracrine effects like follicular growth and oocyte maturation, and endometrial receptivity. Therefore, the intrafollicular hormonal environment not only dictates the quality of the developing oocyte but also determines the systemic endocrine conditions required for successful implantation.

Pharmacological inhibition of aromatase has emerged as an attractive strategy to modulate this balance [2, 3]. Aromatase inhibitors, such as letrozole (LTZ), were originally developed for the treatment of estrogen receptor–positive breast cancer but have also been utilized as a co-treatment in breast cancer patients undergoing OS for fertility preservation [4, 5].

In infertility treatment, LTZ is used as first-line treatment to induce ovulation in type 2 anovulatory women undergoing insemination treatment [6, 7]. In recent years, LTZ has been introduced as co-treatment during OS, with the dual rationale of transiently increased intraovarian androgens to enhance follicular sensitivity to follicle-stimulating hormone (FSH) by upregulating FSH receptor expression and promoting early follicular growth, as well as reducing gonadotropin consumption during treatment. It has also been hypothesized that LTZ may improve endometrial receptivity, but studies have not yet been able to show a positive effect on implantation and pregnancy rates [8].

While androgens have been shown to be beneficial for early follicular phase follicle development [9, 10], the role of androgens in preovulatory follicle development remains uncertain. Excessive androgen concentrations in preovulatory follicles have been hypothesized to impair GC function and compromise oocyte competence [11]. In this context, effects of LTZ on intrafollicular steroid profiles at oocyte pickup (OPU) and subsequent reproductive outcomes remain poorly defined.

Clinical studies have primarily focused on systemic endocrine changes and treatment outcomes, whereas direct analyses of follicular fluid (FF) of women treated with LTZ are limited. One study found that FF E_2_ was reduced and testosterone increased but did not report an association to clinical outcome [12]. Another study found that testosterone levels were significantly increased and E_2_ significantly reduced in follicles exposed to LTZ [13] but did not assess pregnancy outcomes.

It is well documented that circulating E_2_ concentrations are markedly reduced during LTZ administration, but it is unclear whether intrafollicular E_2_ is suppressed to the same degree. Moreover, LTZ increases luteinizing hormone (LH) and FSH secretion by alleviating E_2_-mediated pituitary feedback, which in turn may stimulate theca cells to produce more androgens [14]. This dual effect—peripheral aromatase inhibition combined with increased gonadotropin-driven androgen synthesis—may profoundly alter the follicular microenvironment.

The standard dosage of 5 mg/day of LTZ during OS is largely extrapolated from oncology protocols, without direct evidence of its appropriateness for infertility patients. However, randomized controlled trials have reported mixed results, with some showing no adverse effects on embryo development or pregnancy rates, and others suggesting modest reductions in fertilization or live birth [2]. Whether such discrepancies reflect differences in intrafollicular hormone dynamics, inter-individual variation, or suboptimal dosing strategies remains unclear.

To clarify these issues, we investigated samples collected in connection with the RIOT (Reducing the Impact of Ovarian sTimulation) studies [14–16]. The RIOT-B study was conducted as an intensive observational cohort of 31 women, allowing detailed endocrine profiling during a natural cycle, during OS including the luteal phase [14]. The present work is based on the RIOT-B cohort and provides the first comprehensive analysis of intrafollicular LTZ- and steroid concentrations in women receiving LTZ compared with controls.

The specific aims of this study were: (1) to report FF concentrations of LTZ; (2) to determine whether intrafollicular E_2_ concentrations were suppressed by LTZ to the same extent as circulating levels; (3) to assess how intrafollicular androgen and progestin concentrations are altered in response to aromatase inhibition; (4) to explore potential associations between FF steroid composition and clinical outcomes. This study offers novel insight into the endocrine mechanisms underlying LTZ use in assisted reproductive technology (ART) and raises important questions about its optimal application.

## Material and method

### Ethical approval

Informed consent was given in accordance with the Helsinki Declaration II and the study followed the EU Directive on GCP and International Council for Harmonization (ICH)-GCP guidelines. Approval was given from The Scientific Ethical Committee of The Capital Region (Protocol no.:H-15021852), the Danish Health and Medical Agency (EudraCT no.: 2015–005683-41) and the Danish Data Protection Agency. This study was registered at clinicaltrials.gov prior to inclusion (NCT02939898). Participation was voluntary without any economic incentive [14].

#### Study participants

FF samples for this study were collected at the Reproductive Unit, Department of Gynecology and Obstetrics, Herlev Hospital, Denmark, from 2016 to 2018 in women undergoing infertility treatment [14]. For the present analysis, samples from the following cycle were used: Included women underwent OS following a standard antagonist protocol with a fixed daily dosage of recombinant FSH 150 IU (rFSH, Gonal-f®, Merck, Germany) from cycle day (CD) 2–3, and a daily injection of gonadotropin releasing hormone (GnRH) antagonist 0.25 mg (Orgalutran®, MSD, United States) from stimulation day (SD) 5–6. In addition, women were randomized to receive a daily dose of 5 mg LTZ or placebo.

When at least two follicles exceeded 17 mm in diameter, final oocyte maturation was induced with recombinant human chorionic gonadotropin (rhCG) 250 mg (Ovitrelle®, Merck, Germany). OPU was performed 34–36 h after hCG injection. Oocytes were fertilized with partner or donor sperm with in vitro fertilization (IVF) or intracytoplasmic sperm injection (ICSI), depending on sperm quality. Fresh transfer of an embryo was performed at either cleavage stage (CD 3) or blastocyst stage (CD 5) according to standard practice. All patients received luteal phase support for 16 days starting the day of OPU with vaginal micronized progesterone (P_4_) 100 mg (Lutinus®, Ferring, Copenhagen, Denmark) three times a day. Pregnancy was determined by a serum hCG measurement 16 days after OPU, and ongoing pregnancy was determined around 7 weeks of gestation with transvaginal ultrasound showing a viable fetus.

The following exclusion criteria were used: polycystic ovary syndrome (PCOS), hypertension, smoking, anti-Müllerian hormone (AMH) < 4 pmol/L, CD 2–3 FSH > 12 IU/L, previous IVF treatment with less than 4 oocytes or a history of more than 2 treatment cycles without pregnancy [14].

Patient characteristics and outcome of the stimulation cycle are provided in Supplementary Table 1. In total, Forty-five women were included, but seven were excluded before the OS cycle due to hypertension, spontaneous pregnancy, adnexal pathology, or withdrawn consent. An additional eight patients were excluded during the cycle due to lack of oocytes at OPU, no sustainable embryos, non-compliance with the study medicine, ovarian hyperstimulation syndrome (OHSS), premature ovulation in the stimulated cycle, or a natural cycle > 35 days, thus leaving 30 women in the final analysis, 15 co-treated with LTZ and 15 with placebo. Clinical stimulation characteristics and treatment outcomes by randomization group are summarized in Supplementary Table 2, while differences in systemic endocrine and paracrine hormone profiles between the letrozole and placebo groups are presented in Supplementary Table 3.

All women were scheduled to contribute two FF samples (one from each ovary). However, in the LTZ group, one woman contributed only one sample (*n* = 29), and in the control group, two women contributed only one sample (*n* = 28).

#### Collecting of serum and follicular fluid

Serum blood samples were collected at each visit to the clinic and stored at − 20 °C until analysis [14]. OPU was performed in accordance with the standard routine of the clinic. Follicular fluid from the first punctured follicle on each side was subsequently centrifuged at 300 g for 10 min at room temperature to remove contaminating somatic cells, and the supernatant was collected and stored in cryovials at − 80 °C until analysis.

#### Measurement of letrozole

We developed a liquid chromatography-tandem mass spectrometry (LC–MS/MS) method to enable sensitive and selective quantification of LTZ in FF. The chemical reference standard for LTZ was obtained from Sigma-Aldrich (CAS 112809–51-5), and the deuterated analogue (LTZ-d_4_) used as internal standard (IS) was obtained from Cayman Chemical (LTZ-d₄, CAS 1133712–96-5).

Prior to LC–MS/MS analysis, LTZ was extracted from FF samples using Phree™ phospholipid removal, and analysis was performed on a Micromass Quattro Micro mass spectrometer (Waters Corporation, Milford, CA). A detailed description of the analytical workflow, including extraction procedures, LC–MS/MS conditions, and full method validation and quality criteria in accordance with the ICH guideline [17] and European Medical Agency (EMA) [18] bioanalytical guideline, is provided in Supplementary Materials.

#### Liquid chromatography/mass spectrometry analysis of steroids

Sex steroid hormones in FF were quantitated using a previously published protocol [19]. In brief, steroids were extracted using solid-phase extraction and quantified by LC–MS/MS, using a method fully validated and developed by [20]. This method adheres to the ICH guidelines [17] and enables the simultaneous quantification of 20 steroids involved in steroidogenesis. A brief description of the extraction and quantification procedure is provided in the Supplemental Materials.

#### Aromatase kinetic analysis

CYP19A1-catalyzed conversion of testosterone to estradiol was modelled using Michaelis–Menten kinetics with competitive inhibition by letrozole:

$$v=\frac{{V}_{\mathrm{max}}\left[T\right]}{{K}_{m}\left(1+\frac{\left[I\right]}{{K}_{i}}\right)+\left[T\right]}$$

where v = rate of E2 formation, $\left[T\right]$ = testosterone concentration, ${K}_{m}$ = affinity constant for testosterone, ${V}_{\mathrm{max}}$ = maximal aromatase capacity under those conditions. The affinity constant for testosterone was assumed to be 50 nM, and Ki for letrozole was 2 nM [21]. ${V}_{\mathrm{max}}$ was estimated based on granulosa cell CYP19A1 expression and preovulatory FF E_2_ concentrations (≈1000 nM).

#### Data processing and statistical analysis

Chromatographic data for LTZ in FF obtained via LC–MS/MS were processed using MassLynx version 4.1. Chromatographic peaks for steroid analyses were integrated using MultiQuant 3.0 (AB SCIEX).

All chromatographic peaks (LTZ and steroid concentrations) were visually inspected and manually re-integrated when necessary. Concentrations were calculated by quantifying relative to each analyte to its corresponding deuterated internal standard. Samples exceeding the linear range of the standard curves were appropriately diluted and re-analyzed.

FF steroid and LTZ concentrations were analyzed at the follicle level. For analyses involving clinical outcomes, concentrations were summarized at the patient level (mean value per woman) to account for intra-individual variability and non-independence of samples. Statistical analysis was performed using GraphPad Prism 10.2.3 (GraphPad Prism Software Inc., La Jolla, USA). Differences in LTZ and steroid concentrations between groups were assessed using Student’s *t*-test with Welch’s correction. Correlations between steroid concentrations in FF among women receiving LTZ were evaluated using Spearman’s rank correlation analysis. A *p*-value < 0.05 was considered statistically significant. Graphs were generated in GraphPad Prism 10.2.3.

## Results

**Fig. 1:**
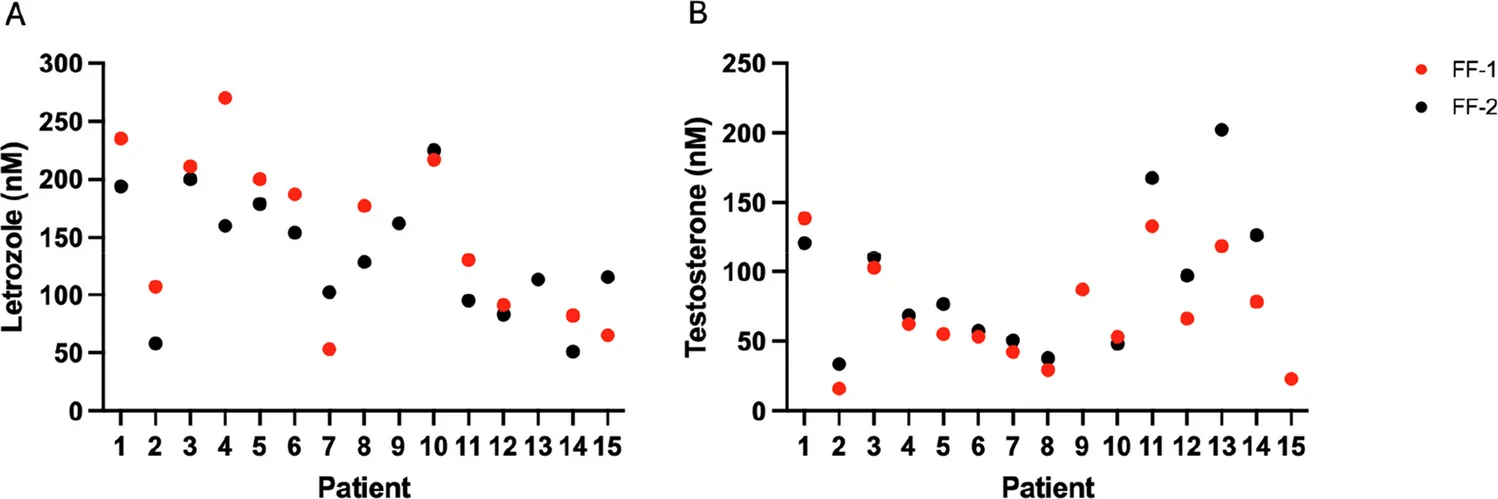
Follicular fluid concentrations of letrozole in individual follicles. Follicular fluid concentrations (nM) of letrozole (**A**) and testosterone (**B**) measured in individual follicles from each patient. For each patient, samples were obtained from two different follicular fluid compartments: follicular fluid 1 (FF-1), shown in red, and follicular fluid 2 (FF-2), shown in black. Each data point represents one individual follicle

The concentration of LTZ in FF was on average 144 ± 12 nM (Mean ± SEM; range 51 to 270 nM). Individual FF samples are shown in Fig. 1, with two samples per patient, demonstrating a high degree of intra-individual concordance for both LTZ and testosterone concentrations.

**Fig. 2:**
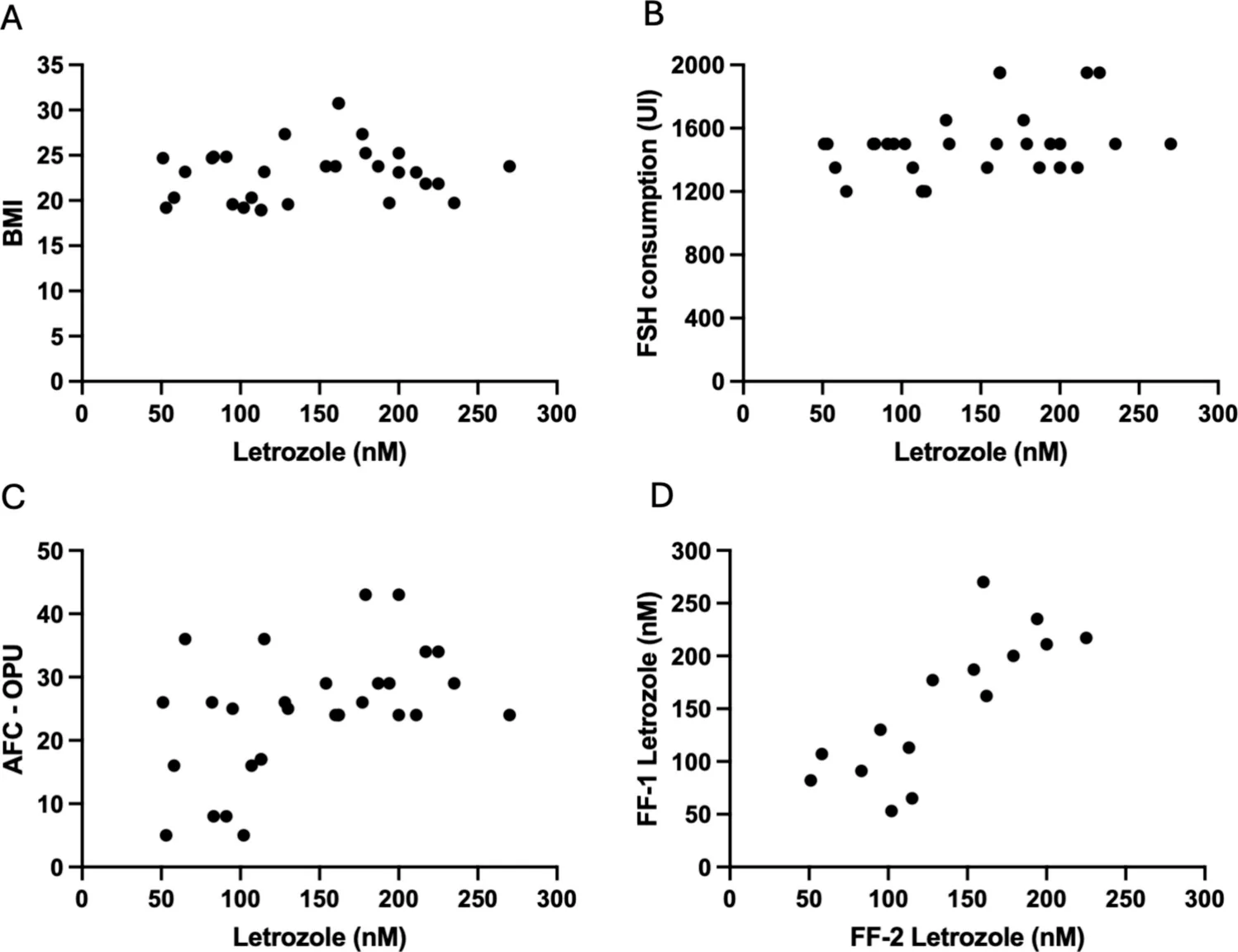
Follicular fluid concentrations of letrozole in relation to BMI, FSH consumption, AFC, and follicular fluid compartments. **A**: Association between body mass index (BMI) and follicular fluid letrozole (LTZ) concentration (nM). BMI is shown on the y-axis and LTZ concentration on the x-axis. **B**: Relationship between total FSH consumption (IU) and follicular fluid LTZ concentration, with FSH consumption on the y-axis and LTZ concentration on the x-axis. **C**: Association between antral follicle count (AFC) at oocyte pick-up (OPU) and follicular fluid LTZ concentration, with AFC at OPU on the y-axis and LTZ concentration on the x-axis. **D**: Correlation between follicular fluid compartment 1 (FF-1; y-axis) and follicular fluid compartment 2 (FF-2; x-axis) LTZ concentrations

No significant associations between the FF concentration of LTZ and BMI or FSH consumption were found, but a significant association between FF LTZ concentration and total number of antral follicles on day of OPU was observed (Fig. 2).

**Fig. 3:**
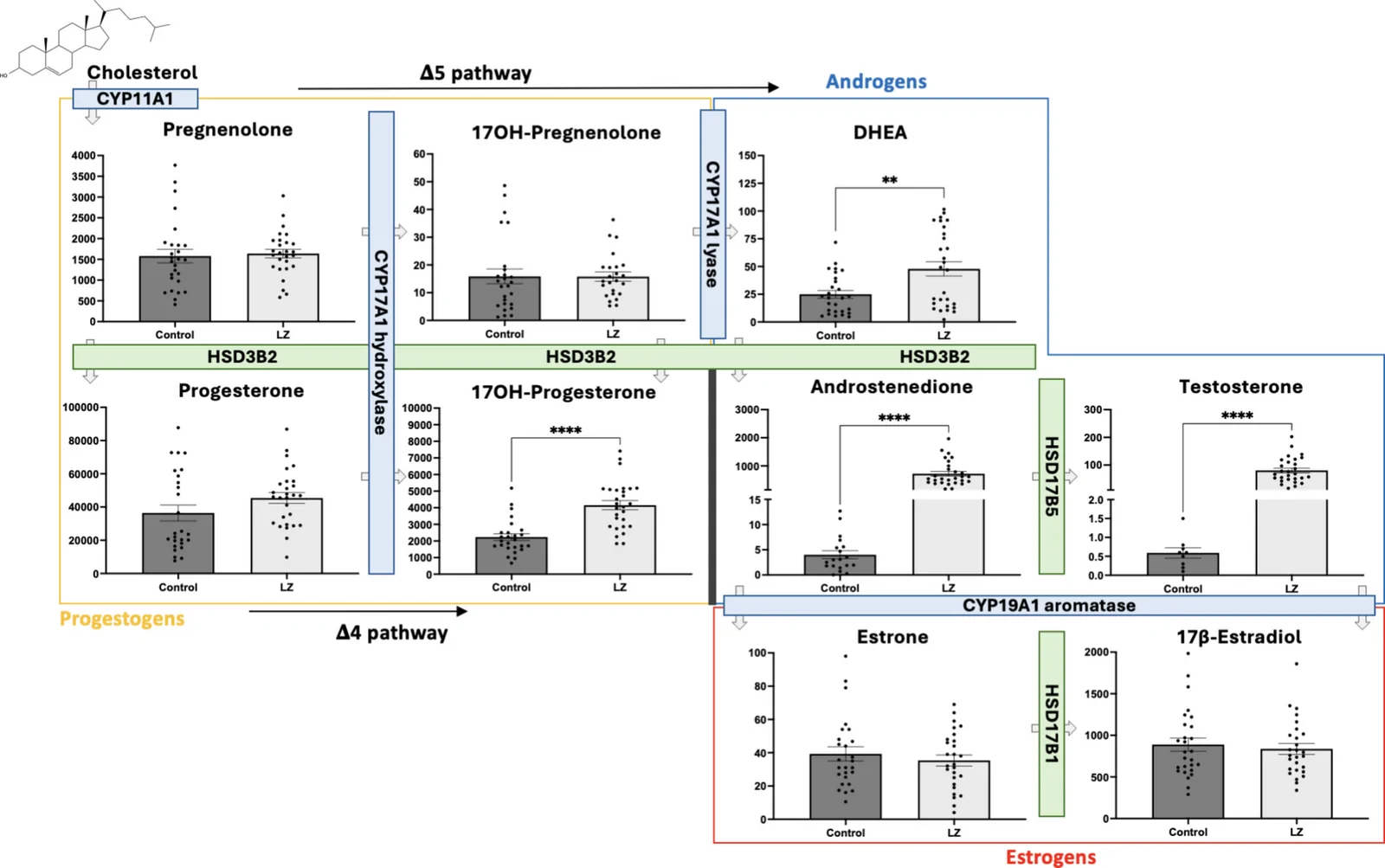
Concentrations of ovarian steroids in preovulatory follicular fluid in relation to letrozole exposure in the control group (dark grey) and the letrozole-treated group (light grey). The Δ5 steroidogenic pathway is shown in the upper panel, and the Δ4 pathway is shown in the lower panel. Enzymes involved in steroidogenesis are indicated in blue for cytochrome P450 (CYP) enzymes and in green for hydroxysteroid dehydrogenases (HSD). Data are presented as mean ± SEM. DHEA denotes dehydroepiandrosterone. Steroid hormone classes are boxed and color-coded as follows: progestogens (orange), androgens (blue), and estrogens (red)

The concentration of ovarian steroids in FF is depicted on Fig. 3 and listed in Table 1. The concentration of E_2_ and estrone were similar irrespective of whether LTZ was administered or not measuring ≈900 nM. In contrast, concentrations of testosterone and androstenedione were massively upregulated with concentrations more than 100 times higher in the LTZ group as compared to the control group. In fact, testosterone concentrations were close to the detection limit in the control group and in more than half of the samples no result was obtained due to low concentrations.

**Table 1:** Steroid concentrations (nM) in preovulatory follicular fluid according to letrozole exposure during ovarian stimulation. Steroid concentrations in preovulatory follicular fluid are presented for women who received letrozole and for control subjects who did not receive letrozole during ovarian stimulation. Data are reported as mean  (SEM) and median [Min; Max]. Group comparisons were performed as indicated, with asterisks denoting statistically significant differences between groups (*p < 0.05; **p < 0.01; ***p < 0.001)

Steroid (nM)		Control group (*n* = 28)	Letrozole (*n* = 29)
Pregnenolone	Mean (SEM)	1579 (165)	1639 (106)
	Median [Min; Max]	1487 [412; 3,767]	1654 [584; 3030]
17OH-Pregnenolone	Mean (SEM)	16 (3)	16 (2)
	Median [Min; Max]	13 [1; 49]	14 [5; 36]
Progesterone	Mean (SEM)	36,401 (4763)	45,398 (3,256)
	Median [Min; Max]	34,359 [7635; 87,753]	46,463 [9799; 86,878]
17OH-Progesterone	Mean (SEM)	2234 (204)***	4153 (276)***
	Median [Min; Max]	2033 [670; 5,183]	4147 [1842; 7,414]
DHEA	Mean (SEM)	25 (4) **	48 (7) **
	Median [Min; Max]	18 [5; 72]	34 [2; 102]
Androstenedione	Mean (SEM)	4 (1)****	723 (84)****
	Median [Min; Max]	4 [0.1; 13]	446 [180; 1,968]
Testosterone	Mean (SEM)	0.6 [0.1] **** (*n* = 9)	81 (9)****
	Median [Min; Max]	0.4 [0.1; 1.5] (*n* = 9)	46 [16; 202]
Estrone	Mean (SEM)	39 (4)	35 (3)
	Median [Min; Max]	22 [11; 98]	18 [4; 69]
Estradiol	Mean (SEM)	890 (80)	915 (100)
	Median [Min; Max]	414 [290; 1,984]	531 [339; 3,019]

The upstream effects on steroidogenesis were reflected by significantly elevated concentrations of the CYP17A1-derived androgens, particularly DHEA, in the LTZ group, indicating increased theca cell steroidogenic activity. While pregnenolone concentrations were high in both groups, levels of its CYP17A1 product, 17OH-pregnenolone, remained relatively low within the Δ5 pathway, consistent with efficient downstream conversion mediated by CYP17A1 in theca cells. In parallel, concentrations of 17OH-P_4_, another CYP17A1-dependent steroid, were significantly increased in the LTZ group, further supporting enhanced theca cell CYP17A1 activity. In contrast, P_4_ concentrations did not differ between groups, indicating that the observed changes primarily reflect upstream theca cell steroidogenesis rather than altered luteal progesterone production (Fig. 3; Table 1). Results of a Spearman correlation analysis between steroid concentrations in FF among women who received LTZ are shown in Supplementary Table 4. The intrafollicular levels of LTZ are highly significantly correlated to the levels of androstenedione, while concentrations of androstenedione, testosterone and E_2_ are highly significantly correlated with one another. Furthermore, 17OH-P_4_ is highly significantly correlated to androstenedione, testosterone and E_2_.

The steroid data following division of patients receiving LTZ administration into two groups, one that conceived and one which did not become pregnant are shown in Table 2.

**Table 2:** Letrozole and steroid concentrations (nM) in preovulatory follicular fluid according to pregnancy outcome. Concentrations of letrozole (LTZ) and selected steroids in preovulatory follicular fluid are presented for women undergoing ovarian stimulation with co-administration of LTZ and stratified by pregnancy outcome. Data are reported as mean (SEM) and median [min; max]. For group comparisons, steroid concentrations were averaged per woman (maximum two follicular fluid samples per woman) and compared between pregnant and non-pregnant women using t-tests. Statistically significant differences are indicated (*p < 0.05)

	Letrozole	Progesterone	17OH-Progesterone	Estradiol	Testosterone	Androstenedione
*Overall (n* = *28 foll., n* = *15 women)*						
Mean (SEM)	144 (11)	45,398 (3197)	4153 (271)	837 (64)	79 (9)	723 (83)
Median [Min; Max]	142 [51; 270]	46,463 [9799; 86,878]	4147 [1872; 7,414]	759 [339; 1,859]	64 [12; 202]	552 [180; 1,968]
*Pregnant (n* = *14 foll., n* = *7 women)*						
Mean (SEM)	118 (14)*	46,455 (4,228)	4123 (482)	814 (71)	53 (9)*	591 (104)
Median [Min; Max]	105 [51; 200]	49,955 [9799; 74,050]	3,29 [1842, 7414]	809 [339; 1323]	48 [12; 127]	449 [188; 1554]
*Non pregnant (n* = *14 foll., n* = *8 women)*						
Mean (SEM)	171 (16)*	44,341 (4,781)	4183 (248)	863 (109)	104 (14)*	856 (119)
Median [Min; Max]	178 [65; 270]	43,329 [21,173; 86,878]	4241 [1844; 5181]	748 [429; 1859]	111 [23; 202]	766 [180; 1968]

**Fig. 4:**
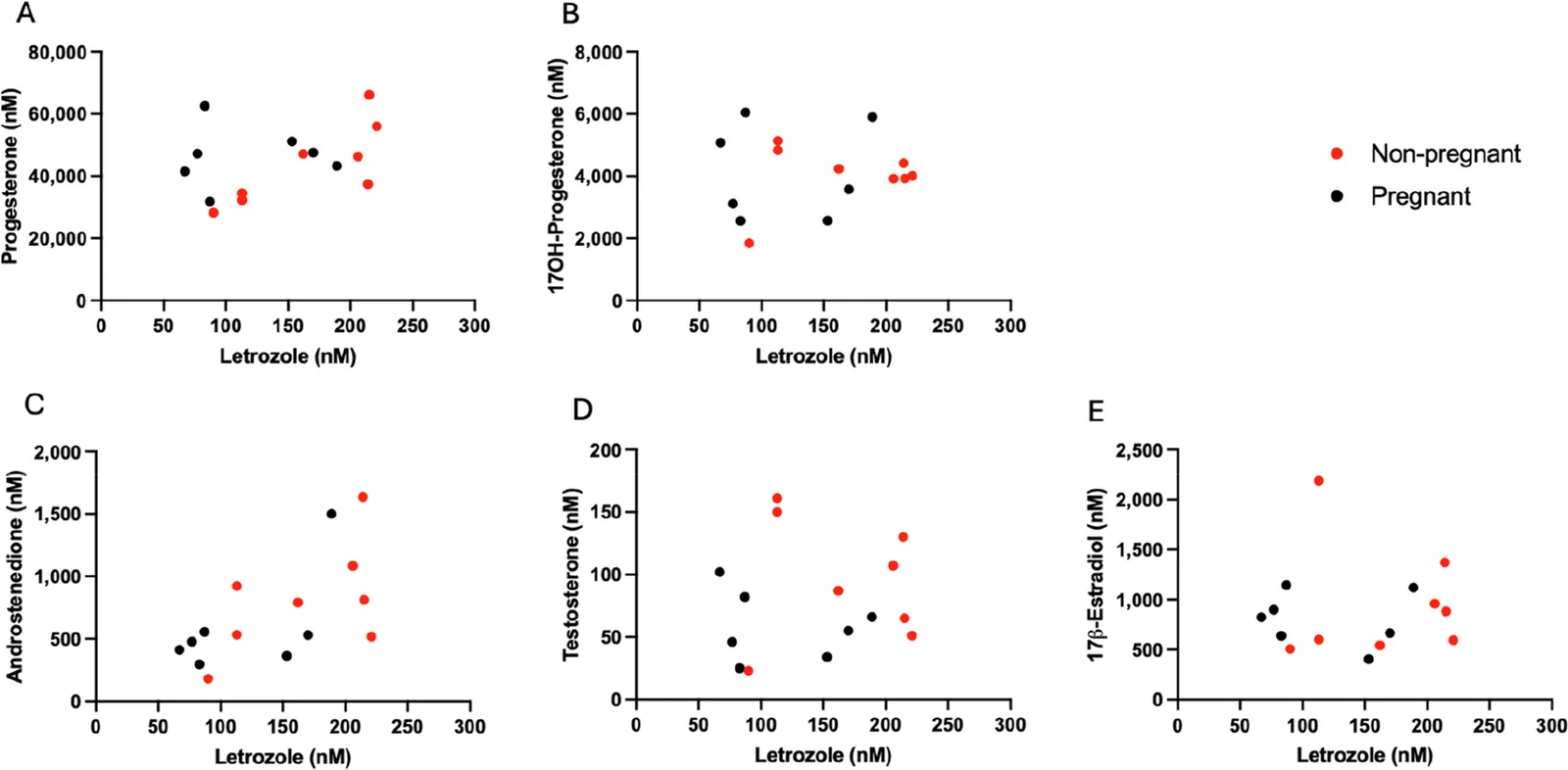
Mean steroid concentrations (nM) in preovulatory follicular fluid in relation to letrozole concentration (nM) according to pregnancy outcome. Each point represents the mean value per woman. **A** Progesterone. **B** 17OH-progesterone. **C** Androstenedione. **D** Testosterone. **E** 17β-Estradiol. Non-pregnant cycles are shown in red and pregnant cycles are shown in black

Two follicles from each woman were included, 8 women failed (a total of 14 follicles were measured) while 7 conceived (a total of 14 follicles were measured). While there were no significant differences in the concentrations of P_4_, E_2_, 17OH-P_4_ and androstenedione as a function of LTZ, we observed a significant difference in the testosterone concentration with higher LTZ concentrations in FF from women who failed to conceive. The relation between LTZ and steroid concentrations and achievement of pregnancy is shown on Fig. 4.

In LTZ untreated follicles, testosterone concentrations fell sharply from ~ 250 nM to ~ 1 nM during the ovulatory period, whereas E_2_ and estrone concentrations remained high (E_2_ ≈ 1000 nM, estrone ≈ 40 nM). In follicles exposed to LTZ, intrafollicular testosterone remained elevated (≈100 nM) despite competitive inhibition of CYP19A1 from LTZ.

Kinetic modelling indicated that the increased substrate concentration effectively compensated for the competitive inhibition (Kmapp ≈ 3.6 µM), resulting in predicted synthesizing velocities of oestrogens similar to untreated follicles (~ 2–3% of Vmax). Accordingly, follicular E_2_ (≈1000 nM) and estrone concentrations were comparable as observed between control and LTZ-exposed follicles confirming the compensation predicted by Michaelis–Menten kinetics.

## Discussion

This study demonstrates that concentrations of E_2_ and estrone in preovulatory FF collected at OPU in a cycle with co-administration of LTZ (5 mg/day) and OS remain similar to that of the control group without LTZ administration. This paradox is explained by competitive kinetics: LTZ markedly increases the apparent K_m_ of CYP19A1 (i.e. ≈ 50 nM → 3.6 µM) (i.e., reducing conversion rate of the aromatase enzyme), while intrafollicular testosterone simultaneously remains at ≈ 100 nM, effectively maintaining aromatase flux of E_2_. In contrast, in the control group without LTZ, levels of androgens in FF become very low (FF-testosterone ≈1 nM; androsterone below ≈ 10 nM reflecting high aromatase activity in the GC compartment exceeding androgen substrate availability resulting in substrate shortage. Calculations using the average measured concentrations of hormone confirm that an almost similar rate of estrogen synthesis takes place in the two situations. Essentially, the equation states that the high E_2_ levels reflect both ongoing synthesis and accumulation within the follicle over hours, emphasizing that enzymatic inhibition does not directly translate to changes in hormone concentrations. Even at testosterone concentrations near 100 nM, aromatase can produce sufficient E_2_ to maintain follicular E_2_ at ≈ 1000 nM. Essentially, the data express that a lower catalytic efficiency (LTZ group) plus much higher substrate result in similar E_2_ output and highlight that in vivo steroidogenesis depends not only on enzyme inhibition but also on substrate availability, enzyme expression, and the kinetic context of product accumulation. The significant associations between androstenedione, testosterone and E_2_ in FF essentially support this notion and underscore the robustness of intrafollicular E_2_ production in the face of pharmacologic inhibition.

The analysis is, however, performed on FF collected at OPU, where intrafollicular E_2_ reaches a nadir, about fourfold lower than FF concentrations before hCG administration [22]. It is possible that a difference in intrafollicular E_2_ concentration exists in the late follicular phase, where E_2_ production is much higher, and where we have previously found a greater E_2_ difference in the systemic concentration between LTZ co-treated women compared to placebo. Since the majority of the circulating E_2_ most likely originates in the preovulatory follicles, a difference in follicular E_2_ in earlier parts of follicular development is likely to happen [23]. However, the overall large systemic differences in E_2_ concentration between LTZ and placebo are also a result of the extraovarian aromatase present throughout the body that also becomes downregulated by LTZ, while the concentrations of androgens, however, are not as vigorously upregulated/accumulated in circulation and do not cause a substrate surplus as compared to the follicle. Thus, it appears that the reduction in circulating levels of E_2_ during LTZ administration is also influenced by a peripheral reduction in E_2_ production. Importantly, the endometrium will be exposed to reduced concentrations of E_2_ reflecting the endocrine conditions in circulation.

Our findings contrast with a previous study that reported reduced concentrations of E_2_ in FF from women with breast cancer undergoing OS with LTZ compared with a control normal group. However, this result does not align with the competitive inhibition of LTZ as predicted by the Michaelis–Menten equations and furthermore steroids were measured using an ELISA technique in contrast to the present study in which the precise LC–MS/MS method was used. Further, the diagnosis may also impact E_2_ synthesis [12].

In contrast, concentrations of androgens were increased in both follicles and in circulation in women exposed to LTZ [14, 15]. The significant accumulation of FF androgens in women from the LTZ group most likely reflects an upstream effect of inhibiting the aromatase enzyme present in follicles and in peripheral tissue, together with increased concentration of gonadotropins in circulation which, via a reduced pituitary feed-back inhibition by E_2_, increased significantly in the LTZ group as compared to the control group [14]. This increase in gonadotropins is likely to have stimulated theca cells androgen production. However, FF androgen concentrations were not increased to similarly high levels as seen in smaller human follicles with diameters from 4 to 16 mm, in which levels were further increased around 3–4 times to 250–300 nM [23].

Data from the current study are based on the results from the RIOT-B, which was part of the larger RIOT-A RCT that included a total of 136 women. The only difference was that women were followed closely during both OS and the luteal phase in the RIOT-B trial [14–16]. The overall endocrine results were similar between RIOT-A and RIOT-B but the larger number of patients in RIOT-A demonstrated that although slightly reduced in LTZ group, oocyte performance, blastocyst formation and pregnancy rates were not significantly affected by the administration of LTZ [16]. The current dogma on the effect on androgens in follicles suggests that androgens are beneficial in small antral follicles but exert negative effects on pregnancy outcomes in preovulatory follicles [24]. However, the high concentrations of androgens in the preovulatory follicles as observed in the current study challenge this dogma and suggest that reduced GC aromatase activity, rather than androgens per se, may be responsible.

However, the current study offers further insight into the action of androgens on folliculogenesis. The RIOT-A study resulted in reduced rates of blastocyst transfer in the LTZ group (30% vs 44%), positive hCG (39% vs 45%), clinical pregnancy rate (31% vs 39%) and live birth rate (28% vs 37%), all non-significant reductions versus the control group. In contrast, the cumulative pregnancy rate (fresh plus FET) increased 4% (non-significant) in LTZ as compared to the control group [15, 16]. These data together with data from the present RIOT-B study showing a pronounced significant difference in FF testosterone concentrations in relation to whether pregnancy was achieved or not are interesting. Women who conceived in the LTZ group showed significantly lower FF levels of LTZ and testosterone, suggesting a systemic effect not specifically related to each individual follicle. Though very small in numbers, it could be hypothesized that androgen and LTZ FF concentrations above a certain threshold exert negative hormonal effects on either the follicle and the enclosed oocyte or on the endometrium to establish a viable pregnancy. However, the number of patients included in this study was too small, despite significant differences, to predict chances of conception highlighting that further data are warranted.

The overall pregnancy rates between groups with and without LTZ remain similar [16] despite an observed negative effect on pregnancy achievement by relatively high FF androgen levels as revealed in the present study. This may reflect two opposing effects, (1) that the reduced circulatory levels of E_2_ plus the enhanced stimulation by endogenous released gonadotropins enhance endometrial receptivity and achievement of conception, (2) which to some extent is counteracted by the negative effects from the high androgen levels. Therefore, further studies on effects of LTZ on pregnancy, for instance based on a reduced dose of LTZ administration or shorter duration, are warranted. The administration of LTZ of 5 mg/day is to our knowledge not founded in actual clinical studies in a group of infertility patients but rather a copy of what is used in oncology. Future studies should focus on the specific LTZ concentrations in FF and in circulation.

17OH-P_4_ concentrations were significantly increased in FF at OPU from women receiving LTZ [14] and highly significant associations to levels in FF of androstenedione, testosterone and E_2_. As 17OH-P_4_ is only produced as a terminal product by theca cells via the Δ4 pathway of ovarian steroidogenesis through the action of CYP17A1 [25], an upstream effect from aromatase inhibition appears to affect steroidogenesis also in the theca cells. In contrast, the concentration 17OH-pregnenolone was low and similar in the two groups, probably reflecting that both the hydroxy steroid dehydrogenase 3B2 (HSD3B2) and the CYP17A1 lyase activity secures a conversion to DHEA and to 17OH-P_4_, which reach very high concentrations. This illustrates nicely that the CYP17A1 lyase reactivity towards 17OH-P_4_ is very low in human theca cells.

There was a more than fourfold difference in FF LTZ concentrations observed in this study, without any association to BMI and FSH consumption, but a relatively weak but significant positive association to the number of antral follicles. This could reflect that the increased gonadotropin levels observed during the follicular phase in the LTZ group [14] are dependent on the LTZ concentration and therefore result in recruitment of more follicles.

It could be hypothesized that high expression of aromatase in GCs would tend to up-concentrate LTZ in FF. However, there were no significant associations between FF concentrations of LTZ and E_2_. Thus, the concentration of LTZ in FF may be determined by other factors such as liver clearance, which the current study has been unable to define. However, it could be interesting to study a possible association between concentrations of LTZ in circulation and in FF.

The circulatory concentration of E_2_ in connection with administration of LTZ in OS is considerably reduced as compared to the control group [14, 26]. This is the argument for the use of LTZ especially in connection with estrogen-sensitive breast cancer patients undergoing OS for fertility preservation. However, the RIOT-B data clearly showed that the concentration of E_2_ during OS in connection with administration of LTZ is on average almost twice as high as that observed during the natural menstrual cycle of the same patients [14]. Therefore, levels of E_2_ during OS with LTZ, which seldom exceeds administration of more than 5 mg per day, are still supraphysiological and it is difficult to assess a potential impact on the stimulation of E_2_-sensitive malignant cells. However, there are data to suggest that no increased relapse rate is observed in women who receive LTZ compared to those who do not receive any treatment [27].

The present study has some limitations. The FF-concentrations were measured in follicles where the destiny of the oocyte was not monitored, but it is known whether the woman conceived or not. So, the intra-individual variations in FF concentrations may account for some of the variability observed in relation to whether conception was achieved or not. However, as shown on Fig. 1 there was relative consistency between the concentrations of LTZ in the two follicles measured in most women.

In conclusion, the present study reveals unexpected characteristics of LTZ administration on ovarian steroidogenesis during OS. First, intrafollicular concentrations of E_2_ at OPU were unaffected by the administration of LTZ, which is in accordance with competitive inhibition as predicted with the Michaelis–Menten kinetics. In contrast, androstenedione and testosterone were highly upregulated in FF from women receiving LTZ, showing an upstream effect of aromatase inhibition. The results showed a significantly reduced likelihood of conception in women with relatively high levels of LTZ and androgens but on a small number of patients, emphasizing that further studies are warranted. The highly significantly increased FF levels of 17OH-P_4_ in women receiving LTZ suggest that this theca cell-derived steroid is only to a limited degree affected by the lyase activity of CYP17A1. Since 17OH-P_4_ is a specific terminal product of theca cells, this observation can only be explained by a yet undescribed effect of LTZ on theca cells. Collectively, the present results put the use of LTZ for OS in a new light that needs to be considered in detail going forward.

## Supplementary Information

Below is the link to the electronic supplementary material.

Supplementary file1 (DOCX 96 KB)

Supplementary file2 (DOCX 39 KB)

## Data Availability

Will individual participant data be available? No What data in particular will be shared? Data from steroid hormone analysis. When will data be available? Immediately following publication. No end date. With whom? Researcher who provides a methodologically sound proposal. For what types of analysis? Any purpose. By what mechanism will data be made available? Proposals should be directed to bjarne.styrishave@sund.ku.dk.

## Declarations

### Disclaimer

During the preparation of this work the author(s) used ChatGPT 5.2 for language assistance. After using this tool/service, the author(s) reviewed and edited the content as needed and take(s) full responsibility for the content of the published article.

### Attestation statement

Data regarding any of the subjects in the study has not been previously published unless specified.
