# Supplementary material for: Letrozole administration during ovarian stimulation: intrafollicular concentrations, effects on steroidogenesis and pregnancy outcome—a secondary analysis
Source: J Assist Reprod Genet. 2026 May 4;43(7):2209–19. doi: 10.1007/s10815-026-03890-6 (PMC13518731; doi:10.1007/s10815-026-03890-6)
Supplement: Supplementary file 1 [file 10815_2026_3890_MOESM1_ESM.docx]

**Supplementary Methods and Materials to:**

**Title: Letrozole administration during ovarian stimulation: intrafollicular concentrations, effects on steroidogenesis and pregnancy outcome: a secondary analysis.**

**Development and validation of an LC-MS/MS method for the quantification of letrozole**

*Extraction of letrozole with Phree™ phospholipid removal*

An aliquot of 100 µL of sample, 50 µL of LTZ-d₄ IS (2 ng/µL in MeOH), and 500 µL of 0.1 % formic acid (FA) in acetonitrile (ACN) (v/v) were loaded onto Phree™ cartridges (Phenomenex, 1 mL). Controls and blanks were included in each analytical run. Controls were prepared by adding 50 µL of LTZ standard solution (0.1 ng/µL in MeOH), 50 µL of LTZ-d₄ IS adding it to 500 µL of 100:0.1 ACN:FA (v/v). Blank samples contained 50 µL of LTZ-d₄ IS and 500 µL of 100:0.1 ACN:FA (v/v). Extraction was performed under vacuum at a flow rate of 1 mL/min. The collected extracts were evaporated to approximately 100 µL under nitrogen at 30 °C, reconstituted with 100 µL MeOH and 800 µL 0.1 % FA in LC-grade water (LC-H_2_O), vortexed, transferred to LC vials, and stored at -18 °C pending analysis.

*LC-MS/MS method development for the quantification of letrozole in follicular fluid*

Analysis of LTZ was performed on a Micromass Quattro Micro (Water Coporation, Milford, CA) mass spectrometer. Chromatographic separation was achieved on a Kinetex™ Biphenyl column (2,6 μm; 100 Å; 50 × 2,1 mm; Phenomenex) maintained at 40 °C. Mobile phase A consisted of 80:20:0,1 H₂O:MeOH:FA (v/v/v), and mobile phase B consisted of 100:0,1 MeOH:FA (v/v). The gradient elution profile and LC-parameters, including time-dependent changes in mobile phase B, and flow rate are summarized in Supplementary Table 5.

During analysis, the LC flow was directed to the mass spectrometer only during the LTZ elution window (~ 2 minutes), in order to avoid contamination of the ion source ending at high organic content and subsequent settling time at 5 % molbile phase B ensured stable conditions before next run. Electrospray ionization (ESI) was performed in positive mode, and the MS source and MRM parameters for LTZ and LTZ-d4 are summarized in Supplementary Table 6 and 7.

These transitions were established by infusing a 10 ppm LTZ solution in MeOH into the flow from the HPLC. The infusion flow was 10 µl/min and the HPLC-flow was 300 µl/min at 50 % mobile phase B. The transitions are consistent with the literature (27).

*Spike-recovery of letrozole in follicular fluid*

Absolute and relative recovery of LTZ were evaluated using 2 and 10 ng spike concentrations, employing both pre- and post-spike protocols. For absolute recovery, 2 and 10 ng of LTZ and 100 ng of LTZ-d₄ were added to 100 µL of blank FF either before extraction (pre-spike) or after Phree™ extraction (post-spike). For relative recovery, LTZ was spiked prior to extraction, while LTZ-d₄ was added post-extraction. The post-spike samples were used as the reference, with analyte response normalized to the IS signal. All samples were analyzed using the developed LC-MS/MS method described above. Recovery percentages were calculated according to Equations 1 and 2:

$Absolute recovery \left( \% \right)=\frac{\frac{\left( {Area}_{pre-spiked analyte} \right)}{\left( {Area}_{pre-spiked IS} \right)}}{\frac{\left( {Area}_{post-spiked analyte} \right)}{\left( {Area}_{post-spiked IS} \right)}}\times100$	(1)

$Relative recovery \left( \% \right)=\frac{\frac{\left( {Area}_{pre-spiked analyte} \right)}{\left( {Area}_{post-spiked IS} \right)}}{\frac{\left( {Area}_{post-spiked analyte} \right)}{\left( {Area}_{post-spiked IS} \right)}}\times100$	(2)

*Letrozole Calibration curve*

A five-point calibration curve (0.25-10 ng/mL) was generated from calibration standards in LC-H_2_O mixed with 50 µL of LTZ-d₄ (2 ng/µL). Standards were analyzed in triplicate. Signal-to-noise (S/N) ratios were determined in QuanLynx V4.1 (Waters Corporation, Milford, MA) by comparing the peak intensity of the four lowest standard concentrations (0.25–1.0 ng/mL, measured in sextuplicate) with a representative baseline noise region of equal integration length. The lowest concentration with an S/N ratio ≥ 10 was considered quantifiable. For this concentration, the standard deviation (SD) of the sextuplicate IS-normalized area ratios was calculated. The slope ($a$) of the corresponding calibration curve, along with the SD, were then used to determine the lower limit of detection (LLOD; equation 3) and lower limit of quantification (LLOQ; equation 4), in accordance with the International Council for Harmonisation (ICH) Q2(R2) guideline (17). The standard curve for LTZ is shown in Supplementary Figure 1.

$LLOD=3.3*\frac{SD}{a}$	(3)

$LLOQ=10*\frac{SD}{a}$	(4)

**Supplemental figure 1:** Calibration curve for letrozole (LTZ) showing the relationship between LTZ concentration (ng/mL) on the x-axis and the peak area ratio of LTZ to internal standard (IS) (area (analyte)/area (IS)) on the y-axis.

The curve met European Medicines Agency (EMA) bioanalytical validation criteria (2011) (18), with deviations of ≤ ± 20 % at the LLOQ and ≤ ± 15 % at all tested concentrations, and with ≥ 75 % of calibration points (minimum six) meeting these limits (18).

Method accuracy and precision were evaluated at four concentration levels (0.25, 0.50, 0.75, and 1.0 ng/mL), each analyzed in sextuplicate. Accuracy was expressed as mean percentage recovery relative to the nominal concentration, while precision was expressed as the relative standard deviation (RSD%). The method demonstrated acceptable precision and accuracy at concentrations ≥ 0.50 ng/mL, whereas increased variability was observed at 0.25 ng/mL, supporting the established LLOQ. Full calibration and validation parameters are summarized in Supplementary Table 8.

**Extraction and quantification of steroids**

*Steroid extraction from follicular fluid using solid-phase extraction*

Steroids were extracted from FF using solid-phase extraction (SPE) based on the protocol of Weisser et al. 2016 (20), with minor adaptations. Prior to extraction, samples were thawed on ice and thoroughly vortexed. SPE was performed using Bond Elut C18 cartridges (500 mg, 6 mL, Agilent). Cartridges were preconditioned with 5 mL MeOH followed by 5 mL LC-H₂O at a flow rate of 1 mL/min. After preconditioning, 3 mL H₂O was added to each cartridge, and 100 µL FF was loaded onto the column. Subsequently, 50 µL IS mixture (steroid-dx, 0.1 ng/µL in MeOH) was added.

The IS mixture contained deuterated analogues of the following steroids: d₃-testosterone, d₃-17OH-pregnenolone, d₄-estrone, d₄-pregnenolone, d₅-17β-estradiol, d₆-dehydroepiandrosterone (DHEA), d₇-androstenedione, d₈-17OH-progesterone, and d₉-progesterone. Control samples consisted of 100 µL steroid mix (0.1 ng/µL in MeOH) combined with 50 µL IS; blank samples contained IS only.

Following sample loading, cartridges were topped with 7 mL H₂O and enriched at ~1 mL/min. Wash steps were performed with 9 mL LC-H₂O and 9 mL 75:25 LC-H₂O:MeOH (v/v), applied under vacuum at approximately the same flow rate. Steroids were eluted using 7 mL 20:80 LC-H₂O:MeOH (v/v). Eluates were evaporated under nitrogen at 60 °C to a final volume of 700 µL, after which 100 µL MeOH was added, and samples were transferred to 1.5 mL LC vials. LC-H₂O was added to a total volume of 1 mL. Extracts were stored at -18 °C until analysis.

For samples requiring dilution, a 1:100 dilution of FF was prepared by mixing 50 µL sample with 4950 µL LC-H₂O. From this dilution, 100 µL was applied to each cartridge and processed identically to undiluted samples.

*Quantification of steroids by LC-MS/MS*

Steroid quantification was performed using an online SPE–LC-MS/MS method as described by Weisser et al. (20). The analytical system comprised an Agilent 1290 Infinity binary pump and an Agilent 1100 HPLC pump, which facilitated both sample clean-up and chromatographic separation. For online SPE, a C18 enrichment column (3.9 × 20 mm, 10 µm) was used to retain steroids and remove matrix components.

Each sample (100 µL) was introduced through a 0.3 µm in-line filter onto the enrichment column. Purification was achieved by washing with LC-H₂O/MeOH/FA (80:20:0.1, v/v/v) at 1 mL/min for two minutes. Chromatographic separation was then performed using a binary gradient with mobile phase A (LC-H₂O/FA 100:0.1, v/v) and mobile phase B (100% MeOH). The MeOH content was increased from 10% to 99.5% over 14 min at a flow rate of 0.300 mL/min.

Detection of steroid analytes was carried out on an AB SCIEX 4500 QTRAP mass spectrometer equipped with atmospheric pressure chemical ionization (APCI). Data acquisition and quantification were performed using Analyst software (version 1.6.2).

Samples exceeding the upper calibration limit were diluted according to the procedure described above. Method performance characteristics—including accuracy, linearity, sensitivity, selectivity, precision, matrix effects, and stability—were consistent with established validation standards.
