# Supplementary material for: Letrozole administration during ovarian stimulation: intrafollicular concentrations, effects on steroidogenesis and pregnancy outcome—a secondary analysis
Source: J Assist Reprod Genet. 2026 May 4;43(7):2209–19. doi: 10.1007/s10815-026-03890-6 (PMC13518731; doi:10.1007/s10815-026-03890-6)
Supplement: Supplementary file 2 [file 10815_2026_3890_MOESM2_ESM.docx]

**Supplementary Tables to:**

**Title: Letrozole administration during ovarian stimulation: intrafollicular concentrations, effects on steroidogenesis and pregnancy outcome: a secondary analysis.**

**Supplementary Table 1. Baseline demographic and clinical characteristics by treatment group.** Baseline characteristics are presented for women randomized to letrozole or placebo. Continuous variables are reported as mean (SD) and compared between groups using independent two-sample t-tests. Categorical variables are reported as number (%) and compared using chi-square tests. Anti-Müllerian hormone (AMH) represents the most recent measurement obtained prior to study inclusion. AFC = Antral follicle count.

|  | **Letrozole (n = 15)** | **Placebo (n =15)** | **P-value** |
| --- | --- | --- | --- |
| **Age (years)** |  |  |  |
| Mean (SD) | 31.7 (3.92) | 31.8 (3.51) | 0.913 |
| **BMI (kg/m2)** |  |  |  |
| Mean (SD) | 23.3 (3.46) | 22.6 (3.64) | 0.551 |
| **AMH (nmol/L)** |  |  |  |
| Mean (SD) | 19.1 (7.80) | 16.5 (6.02) | 0.253 |
| **Total AFC** |  |  |  |
| Mean (SD) | 20.4 (7.16) | 19.9 (5.59) | 0.822 |
| **Infertility duration (months)** |  |  |  |
| Mean (SD) | 27.8 (15.9) | 25.2 (11.5) | 0.566 |
| **Average cycle length (days)** |  |  |  |
| Mean (SD) | 29.0 (2.02) | 28.4 (1.94) | 0.313 |
| **Primary infertility** |  |  |  |
| Yes | 17 (89.5%) | 19 (95.0%) |  |
| No | 2 (10.5%) | 1 (5.0%) | 0.963 |

**Supplementary Table 2. Clinical stimulation and treatment outcomes by treatment group.** Clinical stimulation and outcome parameters are presented for women randomized to letrozole or placebo. Continuous variables are reported as mean (SD) and compared between groups using independent two-sample t-tests. Categorical variables are reported as number (%) and compared using Fisher’s exact test. AFC denotes antral follicle count, and OPU denotes oocyte pickup.

|  | **Letrozole (n = 15)** | **Placebo (n = 15)** | **p-value** |
| --- | --- | --- | --- |
| **Total FSH consumption (IU)** |  |  |  |
| Mean (SD) | 1500 (212.1) | 1520 (211.1) | 0.794 |
| **Follicles > 12 mm on OPU day (no.)** |  |  |  |
| Mean (SD) | 13.1 (6.0) | 10 (4.0) | 0.103 |
| **Follicles > 16 mm on OPU day (no.)** |  |  |  |
| Mean (SD) | 7.2 (2.8) | 5.2 (2.1) | **0.036** |
| **AFC on OPU day (no.)** |  |  |  |
| Mean (SD) | 23.7 (10.0) | 23.5 (5.4) | 0.958 |
| **Aspirated oocytes (no.)** |  |  |  |
| Mean (SD) | 8.2 (3.5) | 7.8 (3.3) | 0.755 |
| **Patient pregnant?** |  |  |  |
| Yes | 7 (43.8%) | 5 (33.3%) |  |
| No | 9 (56.3%) | 10 (66.6%) | 0.716 |

**Supplementary Table 3. Endocrine and paracrine hormone profiles in plasma by treatment group.**
Hormone concentrations are presented as area under the curve (AUC) values for the follicular and luteal phases and reported as median (interquartile range). Differences are expressed relative to the placebo group. AUC values were log-transformed prior to analysis, and between-group comparisons were performed using independent two-sample t-tests. For hormones showing significant differences at individual sampling time points, additional analyses were conducted using two-way mixed ANOVA on log-transformed concentration data; significant time points are indicated.

*Difference relative to placebo, **p-value for independent t-test between randomization groups on log-transformed AUC values, ***Results from a two-way mixed ANOVA on log-transformed time point concentrations.

|  | **Placebo**  **Median (IQR)** | **Letrozole**  **Median (IQR)** | **Difference*** | **P-value**  **AUC**** | **Significant difference at specific time points***** |
| --- | --- | --- | --- | --- | --- |
| **FSH (IU/L)** |  |  |  |  | SD5 |
| AUC follicular phase | 94.7 (88.4-103.1) | 100.3 (88.9-123.0) | 5.9 % | 0.147 |  |
| AUC luteal phase | 55.2 (40.1-67.1) | 47.1 (41.3-58.0) | -14.6 % | 0.552 |  |
| **LH (IU/L)** |  |  |  |  | 2db_trigger |
| AUC follicular phase | 27.1 (17.9-35.1) | 32.7 (26.4-43.4) | 20.5 % | **0.033** |  |
| AUC luteal phase | 34.3 (26.5-49.4) | 27.8 (17.3-55.4) | -18.9 % | 0.496 |  |
| **17β-Estradiol (nmol/L)** |  |  |  |  | 2db_trigger; Trigger; OPU; OPU3 |
| AUC follicular phase | 14.7 (10.0-19.7) | 5.6 (4.9-7.0) | -61.8 % | **0.000** |  |
| AUC luteal phase | 36.5 (25.3-59.1) | 25.2 (19.3-31.2) | -30.9 % | **0.046** |  |
| **Progesterone (nmol/L)** |  |  |  |  | 2db_trigger; OPU; OPU6; OPU10 |
| AUC follicular phase | 7.5 (3.6-14.4) | 9.7 (5.8-15.9) | 29.2 % | 0.123 |  |
| AUC luteal phase | 2,730.9 (1878.3-3295.7) | 3,692.7 (3086.6-4639.9) | 35.2 % | **0.043** |  |
| **AMH (pmol/L)** |  |  |  |  | ­ |
| AUC follicular phase | 100.0 (82.9-132.9) | 145.5 (80.5-181.7) | 45.5 % | 0.449 |  |
| AUC luteal phase | 193.7 (133.3-244.5) | 187.7 (143.8-231.3) | -3.1 % | 0.739 |  |
| **Androstendione (nmol/L)** |  |  |  |  | Trigger; OPU10 |
| AUC follicular phase | 45.22 (35.7-55.3) | 55.1 (49.6-79.3) | 21.9 % | **0.016** |  |
| AUC luteal phase | 93.5 (81.7-113.9) | 114.4 (96.1-149.7) | 22.3 % | **0.006** |  |
| **Testosterone (nmol/L)** |  |  |  |  | Trigger |
| AUC follicular phase | 9.2 (6.9-12.8) | 12.2 (10.4-15.0) | 33.2 % | **0.013** |  |
| AUC luteal phase | 20.9 (18.6-23.9) | 25.4 (21.2-33.8) | 21.6 % | **0.012** |  |
| **17OH-Progesterone (nmol/L)** |  |  |  |  | ­ |
| AUC follicular phase | 41.5 (23.4-72.8) | 96.5 (51.4-161) | 132.5 % | **0.027** |  |
| AUC luteal phase | 119 (74.6-239) | 182 (73.5-350) | 53.2 % | 0.31 |  |
| **DHEAS (µmol/L)** |  |  |  |  | OPU13 |
| AUC follicular phase | 33.7 (24.0-37.1) | 38.6 (32.5-43.8) | 14.7 % | 0.144 |  |
| AUC luteal phase | 66.4 (57.9-68.7) | 71.6 (65.2-80.5) | 7.9 % | **0.011** |  |
| **SHBG (nmol/L)** |  |  |  |  | OPU; OPU3; OPU6; OPU10; OPU13 |
| AUC follicular phase | 857 (705-1249) | 895 (634-1,091) | 4.4 % | 0.73 |  |
| AUC luteal phase | 3,403 (2551-4,646) | 2,421 (1,523-3,269) | -28.8 % | **0.009** |  |
| **Inhibin A (pg/mL)** |  |  |  |  | SD5; 2db_Trigger; Trigger; OPU; OPU10 |
| AUC follicular phase | 1,980 (1,523-2,599) | 3,040 (1,993.6-4,191) | 53.5 % | **0.023** |  |
| AUC luteal phase | 6,376 (5,231-8,317) | 10,673 (7824-13146) | 67.4 % | **0.011** |  |
| **Inhibin B (pg/mL)** |  |  |  |  | SD5 |
| AUC follicular phase | 6,889 (4,392-9,452) | 10,541.5 (5,094-14683) | 53.0 % | 0.14 |  |
| AUC luteal phase | 2,326 (1,779-2,998) | 2,984.3 (1,629-4804) | 28.3 % | 0.54 |  |
| **PAPPA (ng/mL)** |  |  |  |  | ­ |
| AUC follicular phase | 7.3 (4.9-8.7) | 6.64 (6.0-7.7) | -8.7 % | 0.52 |  |
| AUC luteal phase | 12.8 (7.3-15.7) | 11.6 (9.6-13.3) | -9.0 % | 0.47 |  |

**Supplementary Table 4. Spearman’s rank correlation coefficient between intrafollicular letrozole (LTZ) and steroid hormone concentrations**

|  | **P_5_** | **OH-P_5_** | **P_4_** | **OH-P_4_** | **DHEA** | **AN** | **TS** | **E_2_** | **E_1_** |
| --- | --- | --- | --- | --- | --- | --- | --- | --- | --- |
| **LZ** | **-0.28**  **(0.170)** | **0.44**  **(0.031)*** | **-0.01**  **(0.971)** | **0.040**  **(0.844)** | **0.02**  **(0.921)** | **0.64**  **(<0.001)*** | **0.14**  **(0.502)** | **-0.01**  **(0.947)** | **0.09**  **(0.649)** |
| **P_5_** | **—** | **-0.23**  **(0.2992)** | **-0.08**  **(0.7038)** | **0.45**  **(0.021)*** | **0.47**  **(0.017)*** | **-0.04**  **(0.854)** | **0.28**  **(0.159)** | **0.29**  **(0.154)** | **0.11**  **(0.619)** |
| **OH-P_5_** | **—** | **—** | **0.24**  **(0.242)** | **-0.06**  **(0.765)** | **-0.40**  **(0.049)*** | **0.54**  **(0.006)*** | **0.01**  **(0.979)** | **0.25**  **(0.243)** | **0.02**  **(0.933)** |
| **P_4_** | **—** | **—** | **—** | **-0.04**  **(0.844)** | **-0.44**  **(0.018)*** | **-0.06**  **(0.765)** | **-0.30**  **(0.122)** | **0.08**  **(0.678)** | **0.14**  **(0.495)** |
| **OH P_4_** | **—** | **—** | **—** | **—** | **0.25**  **(0.195)** | **0.58**  **(0.001)*** | **0.65**  **(<0.001)*** | **0.55**  **(0.003)*** | **-0.03**  **(0.892)** |
| **DHEA** | **—** | **—** | **—** | **—** | **—** | **0.02**  **(0.921)** | **0.21**  **(0.281)** | **-0.16**  **(0.420)** | **0.11**  **(0.594)** |
| **AN** | **—** | **—** | **—** | **—** | **—** | **—** | **0.59**  **(<0.001)*** | **0.57**  **(0.002*)** | **0.14**  **(0.498)** |
| **Testo** | **—** | **—** | **—** | **—** | **—** | **—** | **—** | **0.46**  **(0.016)*** | **0.14**  **(0.479)** |
| **E_2_** | **—** | **—** | **—** | **—** | **—** | **—** | **—** | **—** | **0.39**  **(0.044)*** |
| **E_1_** | **—** | **—** | **—** | **—** | **—** | **—** | **—** | **—** | **—** |

**Supplementary Tabel 5.** Gradient elution program and flow parameters used for LC separation of LTZ.

| Time | Mobile phase B | Flow rate |
| --- | --- | --- |
| min | % | mL/min |
| 0.0 | 5.0 | 0.3 |
| 0.5 | 5.0 | 0.3 |
| 5.0 | 95.0 | 0.3 |
| 8.0 | 95.0 | 0.3 |
| 8.5 | 5.0 | 0.3 |
| 15.0 | 5.0 | 0.3 |

**Supplementary Tabel 6.** MS source parameters used for LC-MS/MS analysis of LTZ.

| **MS source parameter** |  |
| --- | --- |
| Capillary voltage (kV) | 3 |
| Source temperature (°C) | 120 |
| Desolvation temperature (°C)  Desolvation gas flow (l/hr)  Cone gas (l/hr) | 450  350  100 |

**Supplementary Table 7**. Multiple reaction monitoring (MRM) transitions and MS parameters used for quantification of LTZ and the deuterated internal standard (LTZ-d₄).

|  | Abbreviation | Mw | Parent | Daughter | Dwell | Cone | Coll Energy | RT |
| --- | --- | --- | --- | --- | --- | --- | --- | --- |
|  |  | g/mol | m/z | m/z | Sec | Volt | eV | min |
| Letrozole ion 1 | LTZ | 285.3 | 286.1 | 190.2 | 0.3 | 15 | 34 | 7.14 |
| Letrozole ion 2 |  |  | 286.1 | 217.3 | 0.3 | 15 | 16 | 7.14 |
| Letrozole-d4 ion 1 | LTZ-d_4_ | 289.3 | 290.1 | 194.2 | 0.3 | 15 | 34 | 7.16 |
| Letrozole-d4 ion 2 |  |  | 290.1 | 221.3 | 0.3 | 15 | 16 | 7.16 |

**Supplementary Table 8.** Calibration curve characteristics, recovery, and accuracy parameters for the LC–MS/MS quantification of LTZ.

| Calibration curve |  |
| --- | --- |
| Slope | 0.016 |
| Intercept | 0.0019 |
| R^2^ | 0.9943 |
| Linear range (ng/mL) | 0.25-10.0 |
| LLOD (ng/mL) | 0.10 |
| LLOQ (ng/mL) | 0.31 |
| Recovery |  |
| Relative recovery (%) | |
| 2 ng/mL | 102 |
| 10 ng/mL | 94 |
| Absolute recovery (%) | |
| 2 ng/mL | 78 |
| 10 ng/mL | 90 |
| Accuracy (%, ± RSD, n = 6) |  |
| 0.25 ng/mL | 98.7 ± 43.6 |
| 0.50 ng/mL | 108.1 ± 5.6 |
| 0.75 ng/mL | 100.7 ± 5.2 |
| 1.0 ng/mL | 117.3 ± 12.8 |
